# LEADERSHIP IN LONG-TERM CARE LEADERSHIP: THE NURSING HOME ADMINISTRATOR’S JOURNEY DURING THE COVID-19 PANDEMIC

**DOI:** 10.1093/geroni/igad104.2048

**Published:** 2023-12-21

**Authors:** Lydia Manning, Carey Peerman

**Affiliations:** Chaffey College, Rancho Cucamonga, California, United States; Radford University, Roanoke, Virginia, United States

## Abstract

The COVID-19 pandemic has impacted long-term care (LTC) leadership and has made us all aware of the preciousness of life and the resilience it takes to keep moving forward during challenging times. This study aimed to understand how long-term care administrators have experienced changes in their leadership abilities and explore what resources they have drawn on to manage the COVID-19 pandemic. A qualitative research design included individual interviews (n = 20) conducted with LTC Administrators serving in leadership roles during the COVID-19 pandemic. The data were subjected to thematic analysis. Three categories related to long-term care administrators’ experiences of leading during the COVID-19 pandemic were identified: (1) changes in leadership behavior and decision-making styles, (2) transformational experiences of leadership during COVID-19, and (c) important lessons learned during a pandemic. We found that LTC leaders have responded to the COVID-19 pandemic by adapting, navigating, and creating new ways to be leaders in an environment of rapidly changing regulations. Findings illustrate how LTC leadership has experienced nursing home and assisted living administrators during the COVID-19 pandemic. There have been innovative changes in care processes, workflows, and responses to regulation. Long-term care leaders have been forced to create new ways to lead, resulting in new leadership styles emerging.

